# A retrospective study on the relationship between delirium and long-term cognitive function in elderly women following cervical cancer surgery

**DOI:** 10.1097/MD.0000000000042120

**Published:** 2025-05-23

**Authors:** Yefei Wang, Yongchao Yin, Zhiqiang Wei, Nan Zhou, Xinyu Yao

**Affiliations:** a Operating Room, Xingtai People’s Hospital, Xingtai, Hebei, China; b Gynecology Department, Xingtai People’s Hospital, Xingtai, Hebei, China; c Anaesthesia Department, Xingtai People’s Hospital, Xingtai, Hebei, China.

**Keywords:** cervical cancer surgery, elderly women, long-term cognitive function, MMSE, postoperative delirium, psychiatric history

## Abstract

This study aimed to investigate the relationship between postoperative delirium and long-term cognitive function in elderly women undergoing cervical cancer surgery, providing insights into the long-term effects of postoperative cognitive alterations. A retrospective analysis was conducted on 120 elderly women (≥60 years) who underwent cervical cancer surgery over the past decade. Patients were categorized into a postoperative delirium group (n = 45) and a non-delirium group (n = 75) based on Diagnostic and Statistical Manual of Mental Disorders, Fifth Edition criteria, with initial screening using the confusion assessment method. Cognitive function was assessed preoperatively, and at 1 year and 3 years postoperatively using the Mini-Mental State Examination (MMSE). Multivariate logistic regression analysis was performed to identify independent predictors of long-term cognitive decline, adjusting for potential confounders such as age, underlying health status, psychiatric history, and psychological distress. Postoperative delirium occurred in 37.5% (45/120) of patients. One year after surgery, MMSE scores were significantly lower in the delirium group (*P* < .05), and this difference persisted at 3 years (*P* < .05). Univariate regression analysis identified postoperative delirium, older age, poorer health status, psychiatric history, preoperative psychotropic drug use, longer hospital stay, pain scores, and psychological distress as significant risk factors for cognitive decline at both time points. Multivariate analysis confirmed that postoperative delirium (*P* < .001), age (*P* < .05), poorer health status (*P* < .05), psychiatric history (*P* < .001), preoperative psychotropic drug use (*P* < .05), and psychological distress (*P* < .001) were independent predictors of long-term cognitive decline. Length of hospital stay and pain scores were not significant in the multivariate model (*P* > .05). Postoperative delirium is a strong and persistent risk factor for long-term cognitive decline in elderly women following cervical cancer surgery. Psychiatric history and psychological distress further exacerbate cognitive deterioration. These findings highlight the urgent need for improved perioperative cognitive assessment and management strategies to reduce the long-term impact of postoperative delirium in this high-risk population.

## 1. Introduction

Cervical cancer, a prevalent malignancy among women globally, remains a prominent concern in women’s health.^[1–3]^ Despite a recent decline in its incidence, cervical cancer continues to threaten the lives and well-being of women.^[4–6]^ With advancements in medical science and technology, treatment options for cervical cancer have evolved, and surgical resection emerged as a primary approach, particularly in cases of early diagnosis.^[7,8]^ However, cervical cancer surgery may lead to various complications, including postoperative delirium.^[9,10]^

Postoperative delirium also referred to as postoperative acute cognitive impairment (POCD), manifests as a transient cognitive dysfunction shortly after surgery, which is characterized by diminished attention, memory, reasoning, and comprehension.^[11–15]^ While several patients typically regain normal cognitive function within weeks following surgery, a subset may endure prolonged cognitive challenges, significantly influencing their quality of life and independence.^[16–20]^ Postoperative delirium not only causes challenges for the patient, but also imposes an increased burden on the healthcare system, including prolonged hospital stays, increased care costs, and the need for family and social support.^[21–24]^ As one of the high-risk groups for cervical cancer, elderly women are particularly vulnerable to surgical risks and postoperative recovery challenges, warranting the necessity of further research.^[25]^ However, there remains limited research perfectly exploring the connection of delirium with long-term cognitive function after undergoing cervical cancer surgery in elderly women. Understanding this relationship is critical to improving the postoperative management and quality of life of older women.

Delirium is a complex neuropsychiatric syndrome characterized by acute and fluctuating alterations in attention, cognition, and consciousness. Its pathophysiology is multifactorial, involving various underlying mechanisms such as inflammation, neurotransmitter imbalances, and perioperative stressors. Surgical trauma and anesthesia can trigger systemic inflammatory responses, leading to neuroinflammation and disruption of the blood–brain barrier. This inflammatory cascade can induce neuronal dysfunction and neurotransmitter imbalances, contributing to the development of delirium.^[26–28]^ Moreover, perioperative stressors such as pain, sleep disturbances, and emotional distress can exacerbate neuroinflammatory responses and dysregulate stress hormone levels, further predisposing patients to delirium.^[29,30]^ Understanding the intricate interplay of these pathophysiological mechanisms is crucial for elucidating the etiology of delirium and developing targeted preventive and therapeutic interventions.

In this retrospective investigation, it was attempted to elucidate the intricate correlation of postoperative delirium with subsequent cognitive function in elderly women undergoing cervical cancer surgery. Through analyzing clinical data spanning the past decade, not only the incidence of postoperative delirium was discerned, but also the notable variations in post-surgery cognitive function were compared. The primary objective of this retrospective investigation is to elucidate the intricate correlation of postoperative delirium with subsequent cognitive function in elderly women undergoing cervical cancer surgery, providing insights into the long-term impacts of postoperative cognitive alterations and guiding improved postoperative care and intervention strategies.

## 2. Methods

### 2.1. Research design

This study was approved by the Ethics Committee of Xingtai People’s Hospital. This study adopted a retrospective survey design and aimed to explore the relationship between delirium and long-term cognitive function following cervical cancer surgery in elderly women. Through a review of clinical records, we assessed the incidence of postoperative delirium and analyzed changes in cognitive function at 1 and 3 years postoperatively. The primary purpose of the study was to provide information on the status of cognitive function after cervical cancer surgery to enhance postoperative care and intervention. The study concentrated on older women who had undergone surgery for cervical cancer within the past decade. Patients’ general information included age, gender, educational level, marital status, basic health conditions, etc.

### 2.2. Eligibility criteria

Inclusion criteria: (a) women aged 60 years and above; (b) undergoing surgical treatment for cervical cancer, including total hysterectomy or trachelectomy; (c) no obvious cognitive dysfunction before surgery; (d) the presence of complete clinical records and follow-up data; (e) consent to take part in the study and sign the informed consent document.

Exclusion criteria: (a) the presence of other obvious cognitive dysfunction, such as Alzheimer disease; (b) receiving surgical treatment for cervical cancer before surgery; (c) severe postoperative complications, hindering postoperative cognitive function evaluation; (d) suffering from other major neurological diseases (e.g., stroke or Parkinson disease).

### 2.3. Grouping

This investigation stratified patients into distinct groups contingent upon the occurrence or absence of postoperative delirium: the postoperative delirium group and the non-delirium group. Cases in the postoperative delirium group comprised those who exhibited symptoms of acute cognitive impairment following surgery, while the non-delirium group consisted of patients who did not manifest such symptoms. Postoperative delirium was diagnosed based on the criteria outlined in the Diagnostic and Statistical Manual of Mental Disorders, Fifth Edition (DSM-5). The confusion assessment method (CAM), a widely recognized and reliable tool for delirium screening, was used as the initial assessment. Patients who screened positive for delirium underwent further evaluation by a multidisciplinary team, including anesthesiologists and geriatric specialists, to confirm the diagnosis according to DSM-5 criteria. In cases where delirium symptoms were ambiguous or difficult to distinguish, a psychiatrist was consulted to provide authoritative confirmation. This approach ensured that all diagnoses adhered to DSM-5 standards, though the absence of psychiatrist involvement in every case may have contributed to the higher reported incidence of delirium. The CAM evaluates 4 key features: acute onset and fluctuating course, inattention, disorganized thinking, and altered level of consciousness. These features were assessed daily during the first week following surgery, and any patients exhibiting symptoms consistent with delirium were classified into the delirium group.

### 2.4. Interventions

This study did not involve any specific interventions. It concentrated on analyzing the occurrence of postoperative delirium and changes in cognitive function through the review of patient’s clinical data. Patients received postoperative care following standard clinical protocols, and no targeted treatments were administered for delirium.

### 2.5. Observational indicators

(a) Incidence of postoperative delirium: the number of patients with acute cognitive impairment symptoms after surgery was recorded, which was expressed as a percentage; (b) cognitive function assessment: the standard cognitive function assessment tools were utilized, such as the Mini-Mental State Examination (MMSE) or Montreal Cognitive Assessment, evaluating patients’ cognitive function before surgery, 1 year after surgery, and 3 years after surgery; (c) postoperative complications record: an attempt was made to record any additional serious postoperative complications experienced by patients, such as infection, bleeding, etc.

### 2.6. Statistical analysis

The data were analyzed using descriptive statistics, *t* tests, Chi-square tests, and multivariate regression analysis. Baseline characteristics were compared between the postoperative delirium and non-delirium groups. Multivariate regression analysis was employed to control for potential confounders, including postoperative chemotherapy, radiation therapy, cancer recurrence, and the use of psychotropic drugs, to isolate the effect of postoperative delirium on long-term cognitive function. In addition, to account for other factors that could affect cognitive outcomes, we adjusted for postoperative pain management, psychological status, and the use of pain medications in the multivariate regression models. The *P* values provided reflect results after controlling for these confounders. A *P* value < .05 was considered statistically significant.

## 3. Results

### 3.1. Preoperative clinical characteristics and their association with postoperative delirium

Table 1 presents a comparison between the postoperative delirium group (n = 45) and the non-delirium group (n = 75). There were no statistically significant differences between the 2 groups in terms of age (65.3 ± 3.1 vs 66.1 ± 2.8 years, *P* = .178), educational level (*P* = .22), or marital status (*P* = .622). Similarly, underlying health status (*P* = .761) and BMI (26.8 ± 3.4 vs 25.7 ± 2.9 kg/m², *P* = .108) showed no significant differences.

**Table 1:** Preoperative clinical characteristics.

Trait	Postoperative delirium group (n = 45)	Non-delirium group (n = 75)	t/*χ*²	*P*
Age (years)	65.3 ± 3.1	66.1 ± 2.8	-1.35	.178
Educational level	University and above: 15 (33.3%)	College and above: 30 (40%)	χ² = 1.5	.22
Marital status	Married: 38 (84.4%)	Married: 62 (82.7%)	χ² = 0.245	.622
Underlying health status (no serious chronic diseases)	42 (93.3%)	68 (90.7%)	χ² = 0.093	.761
BMI (kg/m²)	26.8 ± 3.4	25.7 ± 2.9	1.62	.108
History of psychiatric disorders (preoperative)	Yes: 5 (11.1%)	Yes: 8 (10.7%)	χ² = 0.026	.871
Preoperative use of psychotropic drugs	Yes: 12 (26.7%)	Yes: 20 (26.7%)	χ² = 0.581	.445

Preoperative psychiatric factors, including a history of psychiatric disorders (11.1% vs 10.7%, *P* = .871) and preoperative use of psychotropic drugs (26.7% vs 26.7%, *P* = .445), were also comparable between groups. This provides a valid comparative foundation for subsequent analyses assessing the impact of postoperative delirium on long-term cognitive function.

### 3.2. Postoperative clinical factors and their association with cognitive decline

Postoperative recovery plays a crucial role in long-term cognitive outcomes, particularly in patients experiencing delirium. To explore this relationship, we analyzed key postoperative factors and their potential influence on cognitive decline (Table 2).

**Table 2:** Postoperative clinical factors (factors related to cognitive decline after the onset of delirium).

Trait	Postoperative delirium group (n = 45)	Non-delirium group (n = 75)	t/χ²	*P*
Postoperative chemotherapy	Yes: 30 (66.7%), No: 15 (33.3%)	Yes: 48 (64%), No: 27 (36%)	χ² = 0.567	.452
Postoperative radiation therapy	Yes: 18 (40%)	Yes: 28 (37.3%)	χ² = 0.941	.332
Cancer recurrence	Yes: 8 (17.8%)	Yes: 10 (13.3%)	χ² = 0.221	.638
Length of hospital stay (days)	12.4 ± 2.3	9.8 ± 1.9	6.81	<.031
Pain score	6.3 ± 1.2	4.8 ± 1.0	5.27	<.028
Psychological score	15.2 ± 3.4	10.5 ± 2.7	7.49	<.001
Postoperative complications	–	–	–	–
Infection rate (%)	3%	1%	0.01	.93
Incidence of bleeding (%)	4%	2%	0.01	.94
Incidence of other complications (%)	4%	2%	0.01	.94

No significant differences were found between the delirium and non-delirium groups in postoperative chemotherapy (*P* = .452), radiation therapy (*P* = .332), or cancer recurrence (*P* = .638), suggesting these treatments were unlikely contributors to cognitive decline. However, the delirium group had longer hospital stays (*P* < .031), higher pain scores (*P* < .028), and worse psychological scores (*P* < .001), indicating a more challenging recovery, which may have influenced cognitive outcomes. Postoperative complications, including infection, bleeding, and other adverse events, were comparable between groups (*P* > .05), suggesting they had minimal impact on cognitive function. These findings highlight the role of hospitalization duration, pain, and psychological distress in postoperative cognitive decline, emphasizing the need for improved perioperative management.

### 3.3. Cognitive function assessment

**Figure 1.**
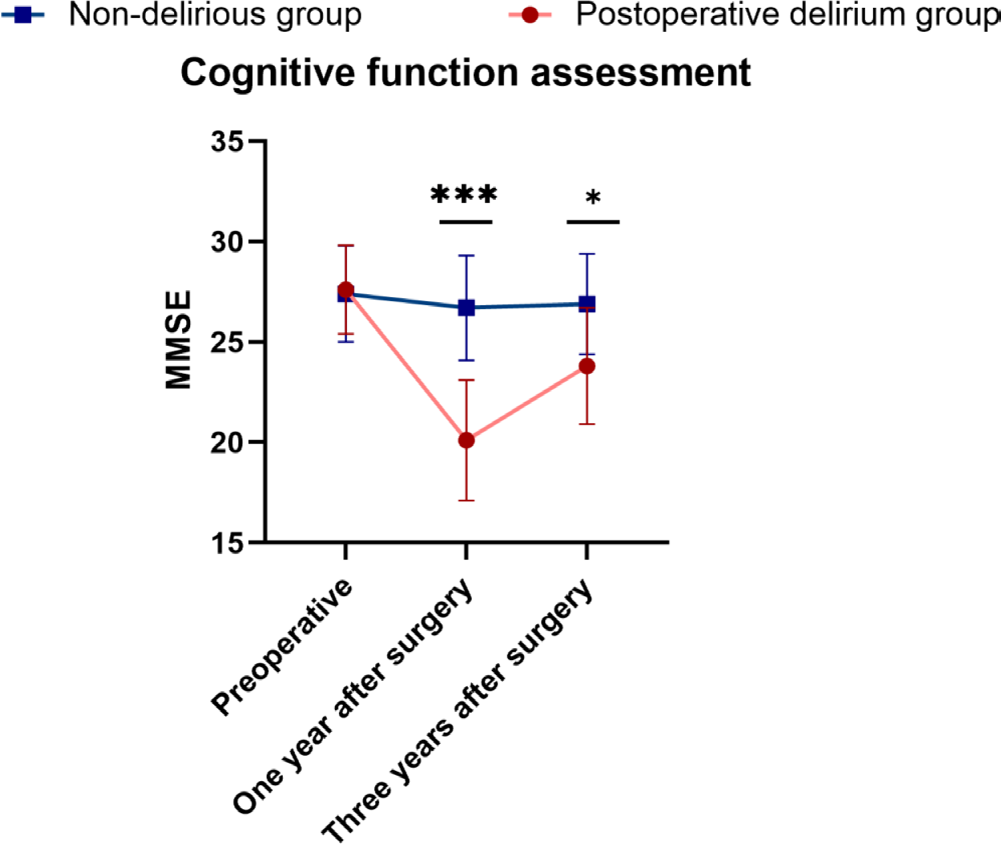
Two groups of cognitive function score change over time line graph *P value between .05 and .01 indicates approaching significance; **P value between .01 and .001 indicates statistical significance; ***P value <.001 indicates high significance with strong evidence to reject the null hypothesis.

Cognitive function was assessed in all patients using standard cognitive function assessment tools. The absence of significant difference, particularly in cognitive function scores, was noteworthy between the 2 groups before surgery. However, cognitive function scores 1 year after surgery unveiled that cases in the postoperative delirium group exhibited a significant reduction relative to the non-delirium group (*P* < .05). Three years after surgery, cases in the postoperative delirium group exhibited cognitive function scores that, while showing improvement, remained notably lower relative to those in the non-delirium group (*P* < .05) (Table 3, Fig. 1).

**Table 3:** Cognitive function assessment results.

Group	Preoperative MMSE (mean ± standard deviation)	Mean ± standard deviation of MMSE 1 year after surgery	Mean ± standard deviation of MMSE 3 years after surgery	t	*P*
Total	27.5 ± 2.3	24.8 ± 3.1	25.6 ± 2.9	-3.82	<.001
Postoperative delirium group	27.6 ± 2.2	20.1 ± 3.0	23.8 ± 2.9	-6.37	<.001
Non-delirious group	27.4 ± 2.4	26.7 ± 2.6	26.9 ± 2.5	2.15	.034

MMSE = Mini-Mental State Examination.

### 3.4. Univariate regression analysis for 1-year postoperative cognitive function

To identify factors associated with cognitive decline 1 year after surgery, we conducted a univariate regression analysis (Table 4). Postoperative delirium (β = -0.72, *P* < .001) was the strongest predictor of cognitive decline. Other significant risk factors included older age (*P* = .003), poorer underlying health (*P* = .011), higher BMI (*P* = .04), psychiatric history (*P* < .001), and preoperative psychotropic drug use (*P* = .031).

**Table 4:** Univariate regression analysis for 1-year postoperative MMSE.

Variable	1-year postoperative MMSE β (SE)	*P*
Postoperative delirium (Yes = 1, No = 0)	-0.72 (0.12)	<.001
Age (years)	-0.19 (0.04)	.003
Educational level (university and above = 1, College and below = 0)	0.28 (0.11)	.025
Marital status (married = 1, Other = 0)	0.10 (0.08)	.21
Underlying health status (no serious chronic diseases = 1, Other = 0)	-0.35 (0.14)	.011
BMI (kg/m^2^)	-0.17 (0.06)	.04
History of psychiatric disorders (Yes = 1, No = 0)	-0.48 (0.16)	<.001
Preoperative use of psychotropic drugs (Yes = 1, No = 0)	-0.25 (0.12)	.031
Postoperative chemotherapy (Yes = 1, No = 0)	-0.22 (0.10)	.018
Postoperative radiation therapy (Yes = 1, No = 0)	-0.29 (0.09)	.009
Cancer recurrence (Yes = 1, No = 0)	-0.15 (0.08)	.048
Length of hospital stay (days)	-0.12 (0.04)	.027
Pain score	-0.36 (0.12)	<.001
Psychological score	-0.42 (0.11)	<.001
Infection rate (%)	-0.01 (0.07)	.89
Incidence of bleeding (%)	-0.01 (0.06)	.94
Incidence of other complications (%)	-0.01 (0.05)	.935

MMSE = Mini-Mental State Examination.

Among postoperative factors, longer hospital stay (*P* = .027), higher pain scores (*P* < .001), and worse psychological scores (*P* < .001) were significantly associated with lower MMSE scores. Cancer-related treatments, including chemotherapy (*P* = .018), radiation therapy (*P* = .009), and recurrence (*P* = .048), also negatively impacted cognition. In contrast, higher education level (*P* = .025) correlated with better cognitive performance, while marital status (*P* = .21) and postoperative complications (*P* > .05) showed no significant effects. These results highlight postoperative delirium, psychological distress, and underlying health conditions as key contributors to long-term cognitive decline.

### 3.5. Univariate regression analysis for 3-year postoperative cognitive function

To evaluate long-term cognitive decline, we performed a univariate regression analysis on MMSE scores 3 years post-surgery (Table 5). Postoperative delirium (β = -0.80, *P* < .001) remained the strongest predictor of cognitive decline. Other significant factors included older age (*P* = .003), poorer underlying health (*P* = .05), psychiatric history (*P* < .001), and preoperative psychotropic drug use (*P* = .045).

**Table 5:** Univariate regression analysis for 3-year postoperative MMSE.

Variable	3-year postoperative MMSE β (SE)	*P*
Postoperative delirium (Yes = 1, No = 0)	-0.80 (0.14)	<.001
Age (years)	-0.25 (0.07)	.003
Educational level (University and above = 1, College and below = 0)	0.30 (0.14)	.05
Marital status (married = 1, Other = 0)	0.10 (0.10)	.21
Underlying health status (no serious chronic diseases = 1, Other = 0)	-0.38 (0.17)	.05
BMI (kg/m^2^)	-0.19 (0.09)	.08
History of psychiatric disorders (Yes = 1, No = 0)	-0.55 (0.19)	<.001
Preoperative use of psychotropic drugs (Yes = 1, No = 0)	-0.27 (0.15)	.045
Postoperative chemotherapy (Yes = 1, No = 0)	-0.20 (0.12)	.06
Postoperative radiation therapy (Yes = 1, No = 0)	-0.28 (0.11)	.055
Cancer recurrence (Yes = 1, No = 0)	-0.18 (0.10)	.08
Length of hospital stay (days)	-0.14 (0.06)	.04
Pain score	-0.42 (0.14)	<.001
Psychological score	-0.50 (0.13)	<.001
Infection rate (%)	-0.02 (0.08)	.895
Incidence of bleeding (%)	-0.01 (0.07)	.94
Incidence of other complications (%)	-0.01 (0.06)	.935

MMSE = Mini-Mental State Examination.

Among postoperative factors, longer hospital stay (*P* = .04), higher pain scores (*P* < .001), and worse psychological scores (*P* < .001) were significantly associated with lower MMSE scores. Cancer-related treatments and recurrence showed weaker associations compared to the 1-year results (*P* > .05). These findings highlight the long-term impact of postoperative delirium, psychiatric history, and psychological distress on cognitive decline, emphasizing the need for continued monitoring and intervention.

### 3.6. Multivariate logistic regression analysis for MMSE

To identify independent predictors of cognitive decline, we performed a multivariate logistic regression analysis on MMSE scores at 1-year and 3-year follow-ups (Table 6). Based on the univariate regression analyses, only variables that were statistically significant at both time points were included in the model to ensure consistency and avoid overfitting. These factors included postoperative delirium, age, underlying health status, history of psychiatric disorders, preoperative use of psychotropic drugs, and psychological score, all of which demonstrated stable associations with cognitive decline.

**Table 6:** Multivariate logistic regression analysis for MMSE.

Variable	Multivariate 1-year postoperative MMSE β (SE)	*P* (1-year)	Multivariate 3-year postoperative MMSE β (SE)	*P* (3-year)
Postoperative delirium (Yes = 1, No = 0)	-0.60 (0.14)	<.001	-0.72 (0.16)	<.001
Age (years)	-0.15 (0.05)	.008	-0.20 (0.06)	.005
Underlying health status (No serious chronic diseases = 1, Other = 0)	-0.30 (0.13)	.02	-0.35 (0.15)	.045
History of psychiatric disorders (Yes = 1, No = 0)	-0.40 (0.17)	<.001	-0.50 (0.18)	<.001
Preoperative Use of Psychotropic Drugs (Yes = 1, No = 0)	-0.22 (0.11)	.028	-0.25 (0.13)	.04
Length of hospital stay (days)	-0.05 (0.06)	.4	-0.06 (0.07)	.38
Pain score	-0.08 (0.07)	.3	-0.09 (0.08)	.28
Psychological score	-0.38 (0.10)	<.001	-0.45 (0.12)	<.001

MMSE = Mini-Mental State Examination.

Postoperative delirium remained the strongest predictor of lower MMSE scores at both time points (β = -0.60, *P* < .001 at 1 year; β = -0.72, *P* < .001 at 3 years). Older age, poorer underlying health, psychiatric history, and preoperative psychotropic drug use were also significant risk factors for cognitive decline over time (*P* < .05). In contrast, length of hospital stay and pain score were not significant in the multivariate model (*P* > .05), suggesting their effects were mediated by other factors. Psychological distress remained a significant predictor of cognitive decline at both time points (*P* < .001), highlighting its long-term impact.

These findings reinforce the persistent influence of postoperative delirium, psychiatric history, and psychological distress on cognitive function, emphasizing the need for early identification and targeted interventions to mitigate long-term cognitive decline.

## 4. Discussion

This study examined the relationship between postoperative delirium and long-term cognitive function in elderly women undergoing cervical cancer surgery. Our findings demonstrate that patients who experienced postoperative delirium exhibited significantly lower cognitive function scores at both 1 and 3 years post-surgery, reinforcing previous research linking delirium to cognitive deterioration. These results suggest that postoperative delirium is not merely a transient complication but a potential contributor to sustained cognitive decline, ultimately affecting patient independence and quality of life.

The strong association between postoperative delirium and cognitive impairment observed in this study supports the hypothesis that delirium may accelerate cognitive decline through neuroinflammatory processes, neurotransmitter dysregulation, and neurodegenerative changes. Neuroinflammation, characterized by microglial activation and the release of pro-inflammatory cytokines, has been implicated in both delirium and long-term cognitive dysfunction.^[31,32]^ Persistent inflammatory responses following delirium episodes may exacerbate neuronal damage and increase the risk of developing neurodegenerative diseases such as Alzheimer disease.^[33–35]^ Additionally, delirium-related alterations in synaptic plasticity and neurotransmitter signaling may disrupt neural circuits critical for cognitive function, leading to sustained deficits.^[36]^ Although the present study did not investigate specific biological markers, our findings align with the growing body of evidence suggesting that postoperative delirium may serve as a precursor to long-term cognitive impairment through these mechanisms. Future research should explore these pathways in greater detail to identify potential targets for intervention.

Beyond postoperative delirium, several other factors were identified as independent predictors of cognitive decline. Older age, poorer baseline health status, psychiatric history, preoperative use of psychotropic drugs, and psychological distress were all significantly associated with lower MMSE scores at both follow-up time points. These findings highlight the interplay between preoperative vulnerabilities and postoperative stressors in shaping long-term cognitive outcomes. Advanced age and preexisting health conditions likely contribute to reduced cognitive resilience following surgery, making these patients more susceptible to postoperative complications, including delirium. The observed impact of psychiatric history and psychotropic drug use further supports the notion that preexisting neurocognitive vulnerabilities may increase susceptibility to postoperative cognitive decline.^[37]^ Moreover, psychological distress emerged as a significant predictor of cognitive impairment, reinforcing the established link between chronic stress, mood disturbances, and cognitive dysfunction. Given the growing recognition of the bidirectional relationship between mental health and cognitive performance, these findings emphasize the need for comprehensive psychological support before and after surgery to optimize recovery.

Among the postoperative factors examined, longer hospital stays and higher pain scores were significantly associated with cognitive decline in univariate analysis but did not remain independent predictors in the multivariate model. This suggests that their impact on cognition may be mediated through postoperative delirium and psychological distress rather than being direct contributors. Interestingly, postoperative chemotherapy, radiation therapy, and cancer recurrence were not significantly associated with long-term cognitive decline in this study. While prior research has linked cancer treatments to cognitive impairment, often referred to as “chemo brain,” our findings suggest that, in this specific cohort, delirium and psychological factors may play a more dominant role in influencing cognitive outcomes. These results underscore the complexity of postoperative cognitive changes and highlight the importance of considering both medical and psychological contributors.

The findings of this study have important clinical implications, particularly for the perioperative management of elderly women undergoing cervical cancer surgery. Given the strong association between postoperative delirium and long-term cognitive impairment, early identification and targeted interventions should be a priority. Implementing delirium prevention strategies, such as optimizing perioperative medication use, employing multimodal pain management approaches, and providing psychological support, may help mitigate the adverse effects of delirium on cognitive outcomes. Additionally, routine cognitive monitoring in high-risk patients could facilitate early detection of cognitive decline, allowing for timely intervention.^[38]^

Compared to the systematic review by Ho et al (2021),^[17]^ our study reported a higher incidence of postoperative delirium. Several factors may have contributed to this discrepancy. First, while our sample size met statistical requirements, it was relatively smaller than that of Ho et al’s review, potentially affecting the generalizability of our findings. Second, our study focused exclusively on elderly women undergoing cervical cancer surgery, and while this population may experience certain age-related physiological changes, hormonal influences, and increased surgical stress, it remains uncertain whether these factors directly contribute to a higher risk of postoperative delirium compared to other surgical populations. Additionally, differences in delirium assessment methods may have influenced the reported incidence. Our study utilized DSM-5 criteria and the CAM, which may have resulted in a higher detection rate compared to studies using alternative diagnostic approaches. Furthermore, variations in patient health status, postoperative medication use, and comorbidities could also explain the higher delirium incidence observed in our cohort. Recognizing these methodological differences is essential for contextualizing our findings and understanding the broader implications of postoperative delirium in different patient populations.

## 5. Limitations

While this study sheds light on the relationship between delirium and cognitive decline following cervical cancer surgery in elderly women, it is essential to acknowledge its limitations. Firstly, as a retrospective study, it relied on clinical records, which may have introduced potential biases and incomplete data collection. Future prospective studies with standardized assessments of delirium and cognitive function could provide more robust evidence. Additionally, the study’s relatively modest sample size may have led to selection bias, limiting the generalizability of the findings and potentially affecting statistical power. Future research should include larger, more diverse cohorts to enhance external validity and ensure sufficient statistical power to detect clinically meaningful differences. Another limitation is that while CAM was used as a screening tool, not all cases were formally diagnosed by a psychiatrist using DSM-5 criteria. Instead, some diagnoses were confirmed by a multidisciplinary clinical team based on CAM findings and clinical judgment, which may have contributed to the higher observed incidence of delirium compared to other studies. Future research should standardize the diagnostic process by ensuring that all cases are confirmed using DSM-5 criteria by a psychiatrist. Furthermore, the absence of specific interventions in this study limits the ability to assess the effectiveness of management strategies for postoperative delirium. Future studies should explore preventive and therapeutic interventions, such as perioperative cognitive rehabilitation and pharmacological treatments, to mitigate delirium’s impact on cognitive function and improve patient outcomes. Addressing these limitations will contribute to a more comprehensive understanding of postoperative delirium and guide improvements in perioperative care.

## 6. Conclusion

In conclusion, an evident correlation emerged between delirium and cognitive decline following cervical cancer surgery in elderly women. This discovery underscored the significance of prioritizing delirium management in postoperative care, highlighting noticeable insights for subsequent interventions aimed at enhancing patient recuperation and quality of life.

## Author contributions

**Conceptualization:** Yefei Wang, Yongchao Yin, Xinyu Yao.

**Data curation:** Yefei Wang, Nan Zhou, Xinyu Yao.

**Formal analysis:** Yefei Wang, Yongchao Yin, Zhiqiang Wei, Nan Zhou, Xinyu Yao.

**Funding acquisition:** Yongchao Yin, Zhiqiang Wei.

**Investigation:** Yefei Wang, Yongchao Yin, Zhiqiang Wei, Nan Zhou, Xinyu Yao.

**Methodology:** Yefei Wang, Yongchao Yin, Zhiqiang Wei, Xinyu Yao.

**Supervision:** Nan Zhou.

**Validation:** Nan Zhou.

**Writing – original draft:** Yefei Wang, Nan Zhou, Xinyu Yao.

**Writing – review & editing:** Yefei Wang, Nan Zhou.
